# News and Events of the Week

**Published:** 1911-01-21

**Authors:** 


### News and Events of the Week.

At an inquest held last week on James Thomas James, M.D., F.R.C.S., aged fifty-four, who was found dead at his residence in Harley Street, Cavendish Square, a verdict of "Suicide while temporarily insane" was returned.

The General Council of the University of Glasgow has approved of a draft ordinance giving effect to new arrangements for medical chairs in the University. These include the foundation by the Muirhead Trustees of two new chairs of Pathology and Obstetrics and the increase in the fees of existing chairs of Clinical Medicine and Clinical Surgery.

On Monday, February 6, Staff-Surgeon Charles Marsh Beadnell, R.N., will deliver a lecture before the Guy's Physical Society, in the Physiological Theatre, on the subject of "Modern Bullet Wounds," at 5 p.m. on that day. Staff-Surgeon Beadnell has seen much active service in the Filipino and South African wars, and his lecture will be illustrated by lantern slides.

For several years Dr. Eden, obstetric physician to Charing Cross Hospital, has been engaged in writing "A Manual of Gynæcology" as a companion volume to his successful "Manual of Midwifery." The object of the book is to present a comprehensive account of the diseases of women from the standpoint of pathology and treatment. The work will be published this month by Messrs. Churchill, and will consist of 750 pages in small royal octavo size. It will contain 300 illustrations, the drawings for which have been specially made for the purpose.

Since the inception of the National League for Physical Education and Improvement in 1905, the question of pure milk has occupied a prominent position in its programme, and a special committee of eminent experts has devoted much time to the problem. The League is now undertaking definite practical steps to deal effectively with the matter. Pending more adequate legislation than at present exists, it is hoped that this action will arouse the nation at large to a sense of its responsibility in the matter. The intention, briefly stated, is to instil into the minds of all concerned in the production and consumption of milk a knowledge of the simple rules required to ensure its purity and cleanliness. Full details of the scheme, and of the standard leaflets on which it is based, will gladly be sent on application to the Secretary of the League, at 4 Tavistock Square, London, W.C.

The annual meeting of the Child-Study Society, London, will be held at the Royal Sanitary Institute, 90 Buckingham Palace Road, on Thursday, March 23, at 6.30 p.m. The next course of lectures and discussions commence on January 26, at 7.30 p.m.: January 26, "Recreational Acitivities of Girls during Adolescence," by Mrs. M. Scharlieb, M.D., M.S.; February 9, "Recreational Activities: English Folk Dances" (with demonstrations), by Cecil J. Sharp, "Training of Gymnastic Teachers," by Lieut. Braae-Hansen; February 23, Discussion: "Should Games be Organised?" March 9, "Appreciative Music Study: Its Application in the Training of the Child," by Stewart Macpherson; March 23, "Educational Possibilities of the Boy Scout Movement," by W. H. Stuart Garnett; April 6, "The Measurement of the Intelligence: With Special Reference to Mentally Defective Children," by A. R. Abelson, D.-ès-L. The Conference of the combined societies will be held at Halifax on July 13, 14, and 15, under the presidency of Sir James Crichton-Browne, LL.D., F.R.S. The subject for discussion will be "Youth (from thirteen to eighteen years) in its Hygienic, Educational, and Social Aspects."

